# Singing and Social Identity in Young Children

**DOI:** 10.3389/fpsyg.2022.823229

**Published:** 2022-06-02

**Authors:** Ioulia Papageorgi, Jo Saunders, Evangelos Himonides, Graham F. Welch

**Affiliations:** ^1^School of Humanities and Social Sciences, University of Nicosia, Nicosia, Cyprus; ^2^Department of Culture, Communication and Media, University College London (UCL) Institute of Education, London, United Kingdom

**Keywords:** young children, singing development, wider benefits, social identity, *Sing Up*

## Abstract

A range of studies suggest that singing activities with young children can have a beneficial impact on other aspects of their development. However, there is little research examining the relationship between young children's singing and their developing social identity. In the current study, data were captured of young children's singing and social identity as part of a larger-scale, longitudinal evaluation of the nationwide *Sing Up* programme in England. Participants were 720 children aged 5-8 years old. The assessment of young children's singing ability employed an established measure and was undertaken individually. With adult support, the children were also asked to complete a simple questionnaire that focused on selected aspects of their social identity, both in general terms and also related to singing. Key themes embraced their attitudes to singing (at home, in school and in informal settings), singer identity (emotional engagement with singing and self-concept), and perceptions of self (self-efficacy, self-esteem, social integration). Comparative data were collected from young children of a similar age outside the programme. Findings suggested that the programme had a positive impact on children's singing ability, both overall and including the youngest children. The data analyses suggest that children could be identified as either “pupils with positive singing identity” or “pupils with less positive, or still developing singing identity.” Overall, pupils with a more positive singer identity—irrespective of *Sing Up*-related experience—tended to report more positive attitudes toward singing at school and other settings, had higher perceived levels of self-esteem and social integration, as well as more positive evaluations of their singing ability. Furthermore, the research suggests that successful participation in high-quality singing activities is likely to have a positive impact on young children's singing ability and, by implication, such positive singing development will also be associated with aspects of self that are related to contexualised singer identity and their sense of social inclusion.

## Introduction

Singing is a commonplace human activity. Recent international ethnographic research (Mehr et al., 2019) indicates that musics—instrumental and vocal—are both universal, yet diverse, human behaviors. Universality is evidenced in reports of music within each of the 315 diverse societies in a global database that was compiled to create a “Natural History of Song” (Mehr et al., 2019). Furthermore, the characteristic acoustic properties of sample songs for expressions of love, healing, infant care (lullabies) and dance were identified above chance by over 29,000 listeners (Mehr et al., 2019, p. 8). This reported global commonality of singing is also evidenced in analyses of music in infant care settings from longitudinal cohort data of 10,000 children in Australia (“Growing Up in Australia”—Williams et al., 2015), and 19,000 children in the UK (the “Millenium Cohort Study”—Moulton, personal communication). These cohort analyses confirm reports from other studies that singing and other musical activities are often an integral feature of pre-school children's lives, both at home and in their local communities, such as reported in Australia (Barrett and Welch, 2021), Turkey (Türkoglu et al., 2020) and in the Chinese diaspora in London (Wu and Welch, 2022).

Vocal music is an everyday feature in maternal communication across cultures, with evidence that this begins pre-birth as part of the mother's early attachment to her unborn child (Cranley, 1981; Trehub et al., 1993; Lecanuet et al., 1995; Woodward, 2019). Responses to such vocalization are observed systematically between the 22nd and 24th week of fetal development, and studies have revealed that the maternal voice is clearly audible in the womb, embracing vowels, timbre, pitch and rhythm (Woodward, 1992, 2019). Across cultures, mothers are observed to sing intuitively to their new-born infants, often in face-to-face contexts, such as to communicate pleasure, or to calm their baby when distressed (Trehub and Cirelli, 2018; Trehub and Gudmundsdottir, 2019). Vocal music is also brought into the home from the wider culture through various electronic media, such as television, tablets, smart phones and children's toys (Young and Wu, 2019). Additionally, infants will experience face-to-face singing outside the home, such as when visiting local “mums and toddlers” groups, or in community-based “music early learning programmes” [MELPS] (e.g., Shehan-Campbell, 2010; Barrett et al., 2019; Barrett and Welch, 2021; Pitt and Welch, 2022).

In addition to this growing body of research into the relative ubiquity of singing in young children's lives, there has been increasing interest in the possible wider benefits of singing. For adults, such benefits have included *inter alia* the experience of psychological wellbeing and a sense of social connectedness as features of the successful membership of adult community choirs and group singing. For example, Bullack et al. (2018) reported social connectedness as likely being related to continued choir membership, a proposition which Glew et al. (2021) suggested has some support in their wider survey of qualitative studies on group singing with children and young people. Camlin et al. (2020) reported on the social bonding effect of group singing, and Clift and Morrison (2011) comment on the benefits of singing in small choirs on mental health. Kreutz (2014) psychobiological study suggests that amateur choral singing enhanced his participant's individual psychological wellbeing and induced a socio-biological bonding response, whilst Pearce et al. (2015) reported that new choral singers experienced a faster sense of social bonding with unfamiliar individuals in comparison with new members non-singing groups, an impact hypothesized as perhaps deriving from the collective nature of choral activity rather than more individualistic nature of crafts and creative writing. Other studies report how participatory music activities can promote verbal learning and recall following stroke in adults and young people (Thompson and Schlaug, 2015; Leo et al., 2018), and to enhance the emotional well-being of new mothers (Fancourt and Perkins, 2018a,b). There is also evidence that effective musical workshop provision for young adults with learning difficulties and/or other disabilities can also bring wider social and emotional benefits, such as related to self-expression, confidence and social skills (Wilson and MacDonald, 2019; MacGlone et al., 2020; Bradford, 2021).

In order to make sense of these findings concerning the potential wider benefits of music making in general, and singing in particular, Savage et al. (2020) synthesize evidence from neuroscience and the social sciences to argue that music's overall evolutionary function is to enable social bonding. They distinguish between human *musicality*—the underlying biological design for creating and making sense of music—and *music—*the diverse cultural products of human musicality. Their neurobiological evidence base draws on our understanding of the interconnected multiple biological mechanisms that facilitate musical perception, production and affective engagement. The authors suggest that “social bonding” is an umbrella term that encompasses a variety of social phenomena, including prosociality. Moreover, our innate human musicality is seen to enhance the potential number of relationships with others (Savage et al., 2020) and is derived from the effects of gene-culture coevolution (*cf* Gintis, 2011), the latter being exampled in the physiology of speech and facial communication, and nurtured in the young through socialization.

There is also evidence concerning the significance of social identity in childhood. For example, Kelly (2009) explored the literature on social identity theories and educational engagement/disengagement and suggested that low academic status is likely to decrease student engagement (p. 459)—a problem that is likely to have been exacerbated most recently by the pandemic and related closure of schools for a significant period. Social inclusion—having a sense of belonging and wellbeing (Faulkner et al., 2020)—is seen as particularly important in an educational context where the focus is on enabling children and young people to access and be successful in their schooling (Rosenberg, 1989; Fredrickson and Furnham, 2004). Moreover, addressing social exclusion in its various guises has been a significant and widespread priority at national and international levels, as exemplified at the 1995 UN World Summit for Social Development (*cf* Atkinson and Marlier, 2010) and, subsequently, with related policy initiatives in the UK (Muijs et al., 2007; House of Lords, 2021), the European Community's initial emphasis on “social cohesion” (European Commission, 1996)—seen as a related concept—and social inclusion (European Commission, 2004), Australia's use of a social inclusion index to measure progress (Faulkner et al., 2020) and in the US State Department's bilateral agreements since 2008 with Latin American countries to support social inclusion and access to opportunity for all (n.d.), as well as within the United Nations (2015). Amongst the evidence of wider benefits associated with socialization and music making are studies of young children experiencing shared music making activities. These may be found in the home, such as related to aspects of increased pro-social skills (Kirschner and Tomasello, 2010; Ilari et al., 2020), as well as in kindergarten (Shen et al., 2019; Ilari et al., 2021). In the Australian longitudinal study mentioned above, for example, parent-child activities in the early years included joint and supported singing, such as action songs, counting songs, nursery rhymes, other children's songs, and original songs to accompany routine activities, as well as dancing, playing basic instruments and listening to music on various electronic media. The higher the frequency of home music activities at ages 2–3 years, the more positive the developmental differences were in children's vocabulary, numeracy, attentional and emotional regulation, and their prosocial skills at ages 4–5 years (Williams et al., 2015). In the UK, not yet published longitudinal data analyses from the Millenium Cohort Study indicate that, after controlling for all co-variates, music at age 3 (“how often they taught their children songs/poems/rhymes…") is associated with higher cognitive ability (naming vocabulary and picture similarity) and fewer reported conduct and peer problems, less hyperactivity and more prosocial behavior and self-regulation at age 5 (Moulton—personal communication). In addition, a recent evaluation of a London-based, professionally mentored singing programme across two school terms with young children aged 6y in a socio-economically deprived inner-city environment revealed statistically significant correlations at the end of the programme compared to baseline between children's singing behaviors and their reading attainment, as well as with aspects of their executive functions related to phonological working memory and inhibition (Welch et al., 2020). Within the literature on self-concept, there are several studies which detail the nature of musical self-concept which is seen to embrace perceptions, beliefs and sense self-efficacy in relation to musical tasks, abilities and potential (Scalas et al., 2017). These perceptions of self are believed to be shaped by experiences of the world and the interpretation of such experiences (Shavelson et al., 1976). Vispoel (1994) and collaborators (e.g., Morin et al., 2016; Scalas et al., 2017) argue that musical self-concept is multi-faceted, being related to the experience of different types of musical activity (such as composing, instrument playing, singing, dancing), and also linked to other aspects of self, such as self-esteem (a sense of general self-worth) and also self-efficacy (how well you believe you can accomplish a task successfully). Furthermore, Scalas et al. (2017) report that with regard to singing in their participant adolescents (ages 11 to 16), higher perceived skills were associated with higher self-esteem.

Overall, there are a range of studies to suggest that singing activities, including with young children, could have a beneficial impact on other aspects of development. Such potential and actual benefits may be particularly important at a time when there is growing official concern amongst Governments about the “attainment gap” in learning between socio-economically disadvantaged children and their relatively better-off peers—a gap which is evident on entry to school and which tends to increase throughout the years of formal education (Andrews et al., 2017; Jerrim et al., 2018). This issue has become more urgent with school closures during the current Covid-19 pandemic, such as reported in the UK (Education Endowment Foundation, 2020) and the USA (Christakis et al., 2020). Consequently, there is a continued concern with ensuring that young children have high quality developmental experiences early in their lives, both in kindergarten and schools (e.g., Stewart and Waldfogel, 2017). Nevertheless, within the available literature, there is relatively little research that has examined the possible impact of engaging in singing activities on the nature and development of young children's wider social identity, notwithstanding the data on pro-social skills in the cohort data reported above and on the links between singing and social inclusion in the earlier report by the current authors (Welch et al., 2014).

### Background to the Study

A four-and-a-half- year, £44 m National Singing Programme, *Sing Up*, was officially launched in England in November 2007 with the intention of ensuring that Primary school-aged children “experience high-quality singing, both within and without their daily school curriculum on a daily basis” and that “Every school has a teacher committed to facilitating high quality singing and vocal work for the whole school.” The rationale being that “Singing offers the most direct route to providing a music-making experience for all children and young people” (2006:8). (For a detailed description of the background and content of the programme, see Welch et al., 2009). By 2012, when the Government's national funding ended, Sing Up was reaching an estimated 98% of Primary schools, embracing 17,000 schools and over 4 m children. Since then, it has continued as a not-for-profit organization that is offered as a subscription service to schools nationally and internationally, with over 60% of Primary schools in England reported as continuing to access the programme through an annual membership fee (https://www.singup.org/about-us). The programme has also been extended to offer resources to support adolescent singing development from age 11 upwards in Secondary schools and the charity offshoot the *Sing Up* Foundation was created in 2017 with a view to promoting singing to support the mental health and wellbeing of children and young people. As part of the funder's evaluation of *Sing Up* after its original launch, a research team from the UCL Institute of Education were appointed to conduct an independent assessment of the effectiveness and impact of the programme. In total, 11,258 children from 184 Primary schools across England had their singing assessed individually over the course of four years. The participant children were also asked to complete a questionnaire which explored aspects of their singing identities and, at the request of the Government, a non-musical measure of children's sense of social inclusion.

### Original Findings From the *Sing Up* Impact Evaluation and Rationale for the Current Follow-Up Analyses

There were several key findings of the programme's impact from the analyses of the collected data. Firstly, in line with the existing research literature on children's singing behavior and development (e.g., Welch et al., 1997; Welch, 2016/2019), (a) older children tended to be rated as more competent in their singing than younger children; (b) girls tended to be more advanced in their singing skills than boys of the same age (Welch et al., 2012); and (c) importantly for the programme's Government sponsors and organizers, based on the first three years of data collection and 11,000+ assessments (Welch et al., 2010), children with experience of the *Sing Up* programme appeared to be, on average, two years in advance developmentally in their singing ability compared to their peers outside the programme. These findings have been revisited below for the whole dataset in the results section as part of a contextualization for the focused analyses of the subset of younger participants.

Secondly, when the analyses of the children's questionnaire data were mapped against the same children's singing assessments, there was a clear positive correlation between singing competency and the children's sense of being socially included for the dataset as a whole (Welch et al., 2014). This relationship was observed to hold both for the children within the *Sing Up* programme, as well as those children without such enriched experiences. In both groups, the more skilled singers tended to report higher levels of social inclusion, whereas less skilled singers were more likely to see themselves as being less socially included. However, a breakdown for children in the youngest age groups was not undertaken and, given the current concerns with young children's wider development, it seemed important to revisit the data to see what relationship might be evident between singing and aspects of social identity for the youngest participants.

In relation to (c) above concerning singing development differences, because the overall dataset was focused more on older children at the request of the funders, separate detailed analyses of the youngest participants were not undertaken. Nevertheless, given the topical importance in addressing the needs of this age phase as outlined in the literature review above, these were the starting points for the current investigation into any specific impact of the *Sing Up* programme on the youngest participants in the dataset. We were also interested to see if the questionnaire analyses also revealed similar trends in terms of young children's developing musical identities, and whether their sense of being socially included was related to assessed singing competency.

### Current Study

The current study sought to investigate further the impact of *Sing Up*, a nationwide intervention programme focusing on delivering high-quality singing experiences for children. More specifically, our investigation aimed to understand the nature of the relationship, if any, between singing competency and children's social identity.

Accordingly, the results section below first provides an overview of singing data across the whole dataset in order to contextualize the singing competency findings with regards the younger children. This is then followed by more detailed analyses of younger children's singing ( ≤ 8 years) and their related questionnaire-based perceptions of singing and social identity.

## Method

### Participants

In total, across the programme evaluation, there were 13,096 assessments of 11,258 children over the course of four years, with some children being assessed more than once as their schools transitioned from being outside to inside the programme. Overall, the ages of the children ranged between 5 and 12 years, with a mean age of 9.23 (SD = 1.32). The prime age focus for the main programme evaluation was on older Primary school children (> 8 years old) because these can be more reluctant singers, especially boys (Welch et al., 2010), and this was expected to be the school age range where the greatest national support for singing might be needed.

Nevertheless, for the purposes of the current article with its early years focus, children ≤ 8 years old have been selected from the dataset, corresponding to *N* = 720 participants. These include pupils in Primary school Year 1 (ages 5–6), Year 2 (ages 6–7) and Year 3 (ages 7–8). Closer inspection of the dataset revealed that there were no Year 1 pupils in the *Non-Sing Up* group (see Table 1), and so (a) a comparative research focus has been undertaken related to the singing data (*Sing Up vs. Non-Sing Up*) for children in school Years 2 and 3, aged 6–8 years, embracing *N* = 670 pupils. The wider dataset (*N* = 720 pupils) has been used to explore issues related to social identity as all the pupils completed this questionnaire phase. The participants were evenly distributed with regard to pupil sex, with 49.4% being female. Participating pupils came from 42 schools spread across the whole of England, and ~73% of the pupils in the dataset were attending a *Sing Up* school at the time of the assessment, i.e., a Primary school that was participating in the Government's National Singing Programme.

**Table 1:** Research participants in school years 1, 2, and 3.

		**Non-Sing Up**	**Sing Up**	**Total**
**Year Group**	**Year 1**	**0**	**50**	**50**
	Year 2	**21**	**168**	**189**
	Year 3	**160**	**321**	**481**
Total		181	539	720

*The statistical analyses of singing in this article are focused on years 2 and 3, N = 670 (highlighted in bold)*.

### Data Collection Instruments

#### Assessment of Children's Singing Ability

In line with the research procedures for the main *Sing Up* impact evaluation, each child had their singing assessed individually in the performance of two well-known songs that are common in a child-focused repertoire—normally singing either “Twinkle Twinkle…” and “Happy Birthday,” or one or other items that the individual child knew well—on advice from their teacher—if these two standard songs were unknown. Developmental singing competency for each of the two focus songs was assessed against two established rating scales (Rutkowski, 1997; Welch, 1998). Previous research (Mang, 2006) had demonstrated that the two scales could be used alongside each other to investigate complementary aspects of singing development. Collectively, the scales offer a holistic perspective of a child's current singing behavior: the Rutkowski (1997) scale is a measure of singing voice development related to vocal register use and vocal pitch range, whereas the Welch et al. (1998) scale assesses vocal pitch-matching development. In the subsequent data analyses, the various scores of each song on each scale were combined and normalized out of 100 (Welch et al., 2011).

#### Assessment of Children's Attitudes to Singing and Perceptions of Self

Pupils' attitudes to singing were investigated with a questionnaire (originally based on Joyce, 2005), which explored children's perceptions in relation to various singing environments (school, home, informal settings), as well as their identity as singers. The questionnaire comprised 60 statements and was structured around eight sub-themes, each of which consisted of a number of statements capturing the issues under investigation in more detail. These sub-themes were as follows, with example items in brackets (the full questionnaire can be found in Welch et al., 2014):

1. Identity as a singer—emotional engagement with singing (e.g., I sing when I am happy);
2. Identity as a singer—self-concept (e.g., I have a good singing voice);
3. Singing at home (e.g., I sing with my family);
4. Singing at school (e.g., I like the songs that I sing at school);
5. Singing in informal settings (e.g. I like singing in the playground);
6. Self-esteem (e.g., I feel good about myself);
7. Self-efficacy (e.g., When I make plans, I think that I can make them work); and
8. Social integration (e.g., I have many friends).

The questionnaire items were organized into three main overarching themes that represented: (a) *singing environments*; (b) *identity as a singer*; and (c) *social inclusion*. These were confirmed by a Principal Component Analyses (Varimax with Kaiser Normalization), both for the whole dataset and also for the subset of younger children's responses. In relation to *singing environments*, children's attitudes were investigated to singing at school, singing at home and singing in informal settings with peers (23 items). With respect to their perceived *identity as a singer*, perceptions were explored about self and emotional connections with singing (22 items). The *social inclusion* questions took account of participants' self-esteem, self-efficacy and perceptions of social integration (15 items). Internal reliability analyses using Cronbach's alpha suggested that the questionnaire as a whole had good internal reliability (α = 0.87). The sub-themes also evidenced satisfactory internal reliability, ranging from 0.60 to 0.80.

Children were requested to indicate their degree of agreement to each of the statements, using a seven-point Likert-type scale from “I don't agree” to “I agree.” “Smiley faces” were used to represent visually the various gradations (level distinctions) and to facilitate the decision-making process, especially for younger children. Where needed, individual children answered the questionnaire with adult support. The sub-theme related statements were presented in a randomized order throughout the questionnaire to control for potential order effects.

Child participants' responses under each identified sub-theme were added together to compose a child's total score (with reversal polarity adjustments for “negative” questions). These overall scores were subsequently used in the statistical analyses reported below.

### Procedure

Children were visited at their schools where their singing and vocal behaviors were assessed in a quiet, familiar space away from the classroom. Each child was taken through the assessment protocol, normally being tested individually within a small group of between two to four of their peers from their class. This allowed the other members of the small group to observe and see what was required, as this had been shown previously to be an appropriate method of accessing better quality responses in children than individual testing alone (cf. Plumridge, 1972; and noting that children's singing assessment is context related, Nichols and Lorah, 2020). To avoid the effects of vocal modeling, no starting pitch was given for the song items and, although the member of the research team provided verbal encouragement to the child, they did not offer any sung prompt (*cf*. as advised by Mang, 2006). All children completed the assessments and none were excluded from the study.

The large numbers of participants necessitated a relatively large research team to undertake the fieldwork. Consequently, to promote reliability in the assessment process, this was undertaken initially by moderation, with members of the research team undergoing initial training on sampled items, then undertaking a school visit in pairs prior to making visits on their own. The validity and ease of use of the assessment protocol was established through a short piloting process prior to commencement of the main data collection (see Welch et al., 2011).

The questionnaire was printed in hard copy and distributed in class, where it was completed by children in the presence of a researcher and class teacher, with support from the class teaching assistant(s) if needed. The researcher gave a brief description of the study, explained the procedure and answered any questions that pupils had prior to the completion of the questionnaire.

In terms of the overall ethical procedures, all participants (headteachers, teachers and pupils) had the purpose of the assessment explained in advance. This was provided in writing to the school using a specially prepared leaflet that was designed to use language in an age-appropriate way for the children. Each child was provided with a copy of the leaflet prior to participation for themselves and their parents, and the school also took responsibility for explaining the research purpose and process, in line with English law where headteachers are empowered to act on behalf of the children's parents, a responsibility that is normally confirmed in writing by the parents/carers at the beginning of each school year. Under our ethical guidelines (based on https://www.frontiersin.org/articles/10.3389/fpsyg.2014.00803/full#B13 (BERA, 2004) and formal ethical approval, we guaranteed anonymity to all participants and told them that they were allowed to withdraw from the assessment process at any time if they felt uncomfortable, for any or no reason. Participation was invited and not compulsory, and individual children could ask to opt out if they did not wish to have their singing assessed.

## Results

### Impact of the Programme on Children's Singing Ability, Both Overall and for the Youngest Pupils

**Figure 1:**
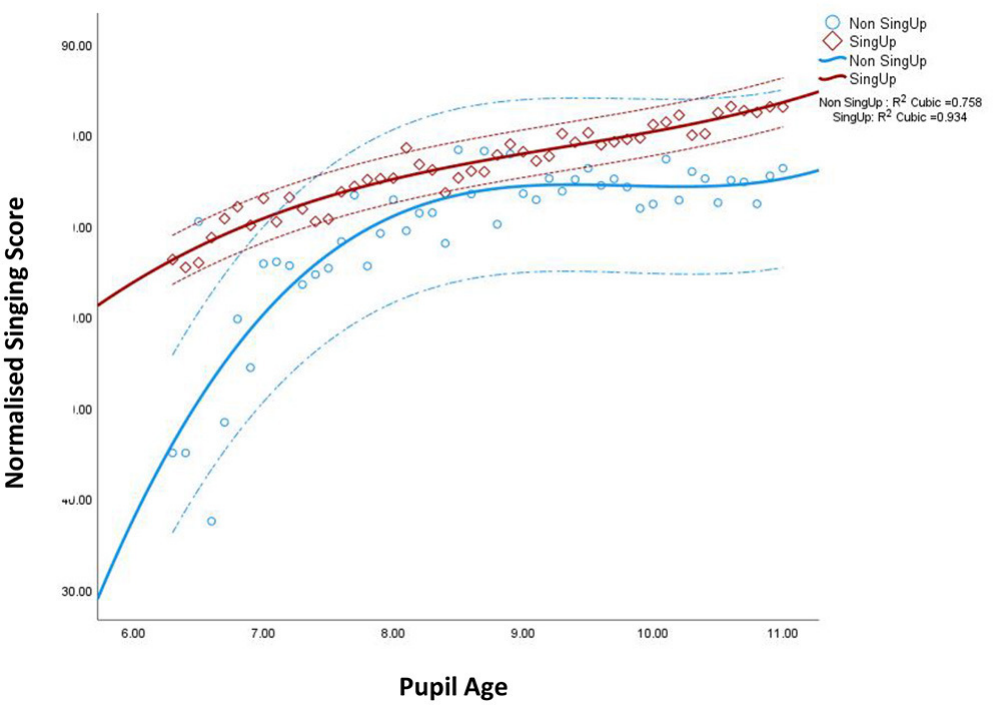
Association between children's mean decimal age and singing competency, normalized across two singing tasks using two rating scales, for participating pupils within and without the *Sing Up* programme, *N* = 11,258 children (*N* = 13,096 assessments); *Sing Up* = 71 schools, *Non-Sing Up* = 56 schools. Data points represent the means for children's decimalised age related to their school grouping (either *Sing Up* and *Non-Sing Up*). Dotted lines represent standard deviations.

When children's mean decimalised age in years, school group and singing are mapped against each other, there is a highly significant correlation across the whole dataset between the decimalised means for age and the normalized singing score (NSS) that is evidenced for each main school status category, *Sing Up r*(69) = 0.934, *p* <0.001 and *Non-Sing Up r*(54) = 0.758, *p* <0.001. The choice of children's decimalised ages had two advantages in that (a) it avoided the visual representation of the distribution being too noisy—as would be the case otherwise with over 13,000 data points, and (b) it enabled the use of a linear age scale to be compared to the linear normalized singing score for the same children. The age-related data in Figure 1 are the means of the normalized singing score for all children in each decimalised age grouping. This comparative data analysis suggests that older children tend to be more advanced in their singing skill compared to younger children and that this effect is evidenced irrespective of whether or not children have experience of the *Sing Up* programme (Figure 1). However, there is a plateau effect in the *Non-Sing Up* data from around the age of 8–9 years.

In general, those participants within the *Sing Up* programme demonstrated a smaller group variance (*Sing Up* M= 77.99, SD = 17.99; *Non-Sing Up* M= 72.72, SD 20.19, and see Figure 1), as well as a trend toward increasing singing competency with age (see Figure 1). In contrast, the *Non-Sing Up* participants appeared to make greater early gain—an artifact of the uneven participant numbers by age (see Table 1)—and then to plateau in mean competency around the age of eight years. This *Non-Sing Up* group also demonstrated greater variation in rated singing for each mean decimalised age (see dotted lines in Figure 1) compared to their *Sing Up* peers.

**Figure 2:**
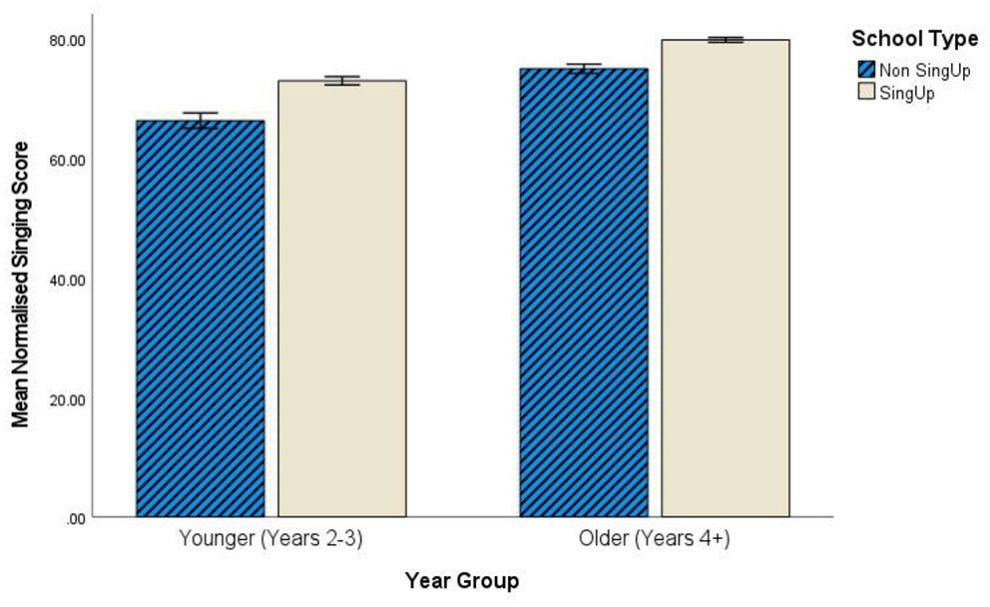
Interaction between age group (school years) and *Sing Up* participation in terms of normalized singing scores.

With regards the main focus for the current article, subsequent statistical analyses confirmed that there was a significant interaction in their normalized singing scores [*F*_(1, 13092)_ = 5.01, *p* = 0.02, partial η^2^ <0.001] between children's age phase (younger = school Years 1–3, ≤ 8 years, versus older = school Years 4–6) and *Sing Up* participation. Further analysis suggests that the effect of *Sing Up* participation was similar in younger children ≤ 8 years (school Years 2–3) as older children (see Figure 2). There were no significant differences evidenced in assessed singing competency between younger children of different ethnic groups (using official school data on younger pupils' ethnicity).

Consequently, a three-way analysis of variance was conducted to investigate the impact of the *Sing Up* Programme, as well as basic demographic variables of sex and school Year group on young children's singing ability. Results for the *N* = 670 pupils in Years 2 and 3 for whom we have both singing data and questionnaire responses revealed significant main effects for *Sing Up* school [*F*_(1, 662)_ = 14.82, *p* <0.001, partial η^2^ = 0.022], pupil sex [*F*_(1, 662)_ = 13.68, *p* <0.001, partial η^2^ = 0.020 ] and Year group [*F*_(1, 662)_ = 14.93, p <0.001, partial η^2^ = 0.022]. Pupils attending *Sing Up* schools evidenced more developed singing ability compared to their peers attending *Non-Sing Up* schools (*Sing Up M* = 78.94, *SD* = 15.71; *Non-Sing Up M* = 71.79, *SD* = 16.17). Findings also suggested that female pupils had higher normalized singing scores (females *M* = 81.49; *SD* = 13.94; males *M* = 72.57, *SD* = 16.94). The same age trend applied to older pupils within this grouping of ages, as Year 3 pupils (*M* = 78.86; *SD* = 16.24) scored significantly higher in singing ability compared to Year 2 (*M* = 72.28; *SD* = 14.92). Taken together, these results suggest that there was a positive impact of the programme on younger participants (as well as older), particularly for females and—within this focus age band—relatively older pupils (Year 3 compared to Year 2).

In order to test for the effectiveness of the programme whilst controlling for the potential effects of the key demographic variables of pupil sex and school Year group—as these had significant main effects on pupils' singing ability—an analysis of covariance was conducted, with singing ability as the dependent variable and with *Sing Up* school as the independent variable, and with pupil sex and Year group entered as covariates in the model. Results suggested that *Sing Up* schools maintained a significant main effect on young children's singing ability [*F*_(1, 666)_ = 37.57, *p* <0.001, partial η^2^ = 0.053] when controlling for the effects of pupil sex and school Year group.

### Impact of the Programme on Young Children's Attitudes to Singing, Singer Identity, and Perceptions of Self

A multivariate analysis of variance was conducted to investigate the impact of the *Sing Up* programme, as well as basic demographic variables of sex and Year group, on the children's questionnaire responses in the eight questionnaire sub-scales. These questionnaire analyses took account of all the young participants (N= 720) in order to provide a more comprehensive view of these young children's perceptions, and allowed an appropriate differentiation by age in the questionnaire data analyses.

Findings demonstrated a significant main effect of *Sing Up* school on self-esteem [*F*_(1, 400)_ = 5.53, *p* = 0.01, partial η^2^ = 0.014]. Results suggested that pupils attending *Sing Up* schools had higher levels of self-esteem compared to pupils attending schools not participating in the *Sing Up* programme (*Sing Up M* = 5.04, *SD* = 1.16; *Non-Sing Up M* = 4.58, *SD* = 1.07).

Analyses also evidenced significant main effects of pupil sex on identity as a singer (emotional engagement) [*F*_(1, 400)_= 16.76, *p* <0.001, partial η^2^ = 0.041], identity as a singer (self-concept) [*F*_(1, 400)_ = 4.51, *p* = 0.03, partial η^2^ = 0.011], singing at home [*F*_(1, 400)_ = 15.28, *p* <0.001, partial η^2^ = 0.038] and singing in informal settings [*F*_(1, 400)_ = 7.37, *p* = 0.007, partial η^2^ = 0.019]. Results suggested that female pupils' identity evidenced more positive emotional engagement with singing (females *M* = 5.76, *SD* = 0.85; males *M* = 5.10, *SD* = 0.11), as well as having a more positive self-concept as singers (females *M* = 5.02, *SD* = 0.79; males M = 4.77, *SD* = 0.78). Female pupils also reported that they sang more at home than their male peers (females *M* = 5.12, *SD* = 1.28; males *M* = 4.43, *SD* = 1.66) and in informal settings (females *M* = 4.64, *SD* = 1.50; males *M* = 4.09, *SD* = 1.62). All means were above the mid-point in the rating scale.

Analyses revealed significant main effects of school Year group on identity as a singer (self-concept) [*F*_(2, 400)_ = 10.04, *p* <0.001, partial η^2^ = 0.049], singing at school [*F*_(2, 400)_ = 3.20, *p* = 0.04, partial η^2^ = 0.016], singing in informal settings [*F*_(2, 400)_ = 6.68, *p* = 0.001, partial η^2^ = 0.033], and social integration [*F*_(2, 400)_ = 9.65, *p* <0.001, partial η^2^ = 0.047]. Findings suggested that the singer identity of older pupils (i.e., Years 2 and 3 in the focus age range) encompassed a more positive self-concept (Year 1 *M* = 4.47, *SD* = 0.84; Year 2 *M* = 5.17, *SD* = 0.99; Year 3 *M* = 4.93, *SD* = 0.87) in comparison with Year 1 pupils. Older pupils (Years 2 and 3) also reported that they sang more at school compared to pupils in Year 1 (Year 1 *M* = 5.65, *SD* = 0.85; Year 2 *M* = 5.29, *SD* = 0.95; Year 3 *M* = 5.38, *SD* = 0.88) and in informal settings (Year 1 *M* = 4.59, *SD* = 1.17; Year 2 *M* = 4.64, *SD* = 1.65; Year 3 *M* = 4.18, *SD* = 1.58), and also felt more socially integrated (Year 1 *M* = 4.56, *SD* = 1.54; Year 2 *M* = 5.48, *SD* = 1.49; Year 3 *M* = 5.14, *SD* = 1.28).

When the analysis was repeated with pupil sex and Year group as covariates in order to explore the effect of *Sing Up* school on the eight subscales, the effect remained significant [*F*_(8, 389)_ = 2.82, *p* = 0.005]. It was further revealed that the programme had a significant and positive main effect, not only on young children's self-esteem [*F*_(1, 400)_ = 9.66, *p* = 0.002, partial η^2^ = 0.024], but also on their attitudes toward singing at home [*F*_(1, 400)_ = 4.50, *p* = 0.035, partial η^2^ = 0.011], and perceptions of social integration [*F*_(1, 400)_ = 4.73, *p* = 0.030, partial η^2^ = 0.012]. Pupils attending *Sing Up* schools had higher mean values in self-esteem (*Sing Up M* = 5.04, *SD* = 1.16; Non *Sing Up M* = 4.58, *SD* = 1.07), more positive attitudes toward singing at home (*Sing Up M* = 5.02, *SD* = 1.48; Non *Sing Up M* = 4.64, *SD* = 1.55), and reported higher perceived levels of social integration (*Sing Up M* = 5.29, *SD* = 1.40; Non *Sing Up M* = 5.01, *SD* = 1.37).

### Impact of the Programme on Profiles of Young Singers

A K-Means cluster analysis, an optimisation clustering technique, was conducted to investigate the presence of profiles of in the young singers' perceptions. Cluster analysis aims at grouping individuals together based on their responses (Bartholomew et al., 2002), and indicates that those allocated together into a cluster share a similar pattern of responses. Data were assessed for outliers, normality, linearity and homoscedasticity of independent variables, and their suitability for K-Means Cluster Analysis was confirmed prior to the analysis. Variables were standardized into z-scores to enable their comparison and to minimize any bias in weighting which may have resulted from differing measurement scales and ranges. Validation of the cluster solution was conducted through Discriminant Analysis. Associations between the emerging clusters and participation in the *Sing Up* programme were investigated using Pearson's Chi-Square Test.

The analysis suggested the presence of two clusters (profiles) of pupils. Cluster 1 included n = 450 pupils, whereas Cluster 2 included n = 270 pupils. Table 2 presents the standardized values (mean = 0, SD = 1) in the key variables for each cluster. As can be seen in Table 2, pupils in cluster 1 had positive (above average) values in the variables; as such, they evidenced a positive singer identity, positive attitudes toward singing at school and other settings, high self-esteem and high levels of perceived social integration. These pupils also had higher singing scores in the assessments of singing ability. In contrast, pupils classified in cluster 2 had negative values in the variables, suggestive of a negative (below average) singer identity, negative (below average) attitudes toward singing at school and other settings, lower self-esteem and lower levels of social integration. These pupils also evidenced lower singing scores in the assessments of singing ability. Cluster 1 was interpreted as “pupils with a positive singing identity” and cluster 2 was representative of “pupils with a less positive, or still developing singing identity.” Results further suggested that identity as a singer was associated with singing ability, as children who reported more positive singing identity were also evaluated as having higher levels of singing ability in their individual singing assessments.

**Table 2:** Final cluster centers.

	**Cluster**	
	**1**	**2**
Identity as singer (emotional engagement with singing)	0.53564	−0.82686
Identity as singer (self-concept)	0.40852	−0.66367
Singing at home	0.49653	−0.75977
Singing at school	0.49807	−0.53606
Singing in informal settings	0.41763	−0.78012
Self-esteem	0.24460	−0.36831
Self-efficacy	0.08957	−0.08284
Social-integration	0.26393	−0.43580
Normalized singing score	0.21075	−0.35125

The effectiveness of the cluster solution was assessed with discriminant function analysis. The two clusters formed the groups for discriminant analysis, and the variables used in the cluster analysis (see Table 2) were used as independent variables. One discriminant function was calculated which had a significant overall Wilk's lambda [Λ = 0.33, χ^2^(9) = 462.55, *p* <0.001].

The discriminant function was successful at distinguishing between the two clusters of students. The function had an eigenvalue of 2.05 and a canonical correlation of 0.82. The η^2^, obtained by squaring the canonical correlation was 0.67, indicating that 67% of the variability of the scores in the discriminant function was accounted for by differences among the two groups of students.

Cluster group membership prediction was also assessed, to investigate how well student group membership could be predicted by using a classification function. Results suggested that 98.5% of cluster 1 and 98.1% of cluster 2 students were predicted correctly. Together, 98.3% of participants were classified correctly. In order to estimate how well the classification functions could predict a new sample, a cross-classification was estimated by selecting the leave-one-out option. Results showed that 97.7% of cluster 1 and 98.1% of cluster 2 students were correctly classified using the leave-one-out classification. Overall, 97.9% of the cross-validated cases were correctly classified.

Pearson's chi-square test was used to assess associations between participation in *Sing Up* and cluster membership. Results revealed no significant association (*p* > 0.05), suggesting that the quality of pupils' singing identity (whether positive or less positive/still developing) was not directly associated with attending a school where the *Sing Up* programme was implemented, but rather more related to their relative individual current singing ability. Variations in singing competency are evidenced in both *Sing Up* and *Non-Sing Up* schools, as evidenced in Figure 1, and so—by inference—membership of each cluster was likely in each school category. Nevertheless, given the mean data finding that children in *Sing Up* schools were likely to be more advanced in their singing (Figures 1, 2), the inference is that the Cluster 2 profile membership is malleable and open to positive experience.

## Discussion

The aim of the current study was to investigate whether *Sing Up*, an ongoing nationwide intervention programme that is focused on providing high-quality singing experiences for children and young people, had a measureable impact on younger children's singing ability, their attitudes to singing and perceptions of self. An evaluation of young children's singing ability was undertaken *via* an established assessment protocol, and children's attitudinal responses were assessed using a self-report questionnaire that focused on their attitudes to singing (at home, at school and in informal settings), singer identity (emotional engagement with singing and self-concept) and perceptions of self (self-efficacy, self-esteem, social integration). The variables were used to compare the experiences of children participating in the *Sing Up* programme and those who were not. Analyses explored the effects of participation in *Sing Up*, as well as key demographic variables of pupil sex and Year group on the aforementioned variables.

### Impact of the Programme on Children's Singing Ability

Findings suggested that children's singing ability tends to improve with age (in general terms), and that the programme had a positive impact on both younger and older participant children's singing ability. At the time of assessment, young children (aged 6–8 years) attending schools participating in the *Sing Up* programme had significantly higher normalized singing scores compared to children outside the programme. The effect was more pronounced in female and relatively older (within this lower age range, school Year 3) pupils, suggesting that these students particularly benefitted from their participation in the *Sing Up* programme. The analyses suggest that the impact of the programme remained significant when controlling for the effects of pupil sex and school Year group, further reinforcing its positive benefits on the development of young children's singing voices. Although there have been reports elsewhere in the literature concerning adults for whom singing behaviors are reported to be normally distributed (e.g., Dalla Bella, 2015; Pfordresher and Larrouy-Maestri, 2015), the evidence here with young children is that any such theoretical distribution is open to change related to age, sex and experience. This evidence of development with age, within and outside the programme, also challenges conceptions of singing as a proxy of musical ability that is relatively fixed (Knight, 2019). The findings here are in line with earlier longitudinal data of improvements in young children's singing competencies and quality with age (Welch et al., 1997; Leighton and Lamont, 2006; Demorest and Pfordresher, 2015—see Welch, 2016/2019 for an overview) and also in relation to effective pedagogy, i.e., pedagogy which nurtures children's engagement with singing and development (Welch, 1986; Fuchs et al., 2006; Siupsinskiene and Lycke, 2011; Hedden, 2012; Pieper et al., 2020).

### Impact of the Programme on Children's Attitudes to Singing, Singer Identity and Self-Perceptions

Analyses indicated that the *Sing Up* programme had a positive impact on young children's self-concept, as children attending schools implementing the *Sing Up* programme reported higher levels of self-esteem. Key demographic variables of pupil sex and school Year group also effected children's attitudes to singing and so the analysis was repeated, controlling for their effects. Results showed that pupils participating in the *Sing Up* programme had higher levels of self-esteem, more positive attitudes toward singing at home and also reported higher levels of social integration. This accords with related findings from the whole *Sing Up* dataset (Welch et al., 2014), as well as empirical studies of the positive effects of singing on identity and well-being with adults (Bailey and Davidson, 2002; Faulkner and Davidson, 2006; Boyce-Tillman, 2019; Davidson and Garrido, 2019) and also children.

### Profiles of Young Singers and Their Association With Singing Ability and Participation in Sing Up

The K-means cluster analysis revealed the presence of two distinct profiles of young singers. The first group, “pupils with positive singing identity,” evidenced positive singer identity, positive attitudes toward singing at school and in other settings, high self-esteem and high levels of social integration. The second group, “pupils with less positive or still developing singing identity,” had more negative (below average) singer identity, more negative (below average) attitudes toward singing at school and other settings, lower self-esteem and lower levels of social integration. Results further suggested that identity as a singer was associated with singing ability, as children who reported more positive singing identity (cluster 1) were also evaluated as having higher levels of singing ability in the singing assessments. The opposite was true for pupils in cluster 2, as they had lower scores in their assessment of singing ability.

This cluster finding provides a complementary narrative to the findings of benefit from *Sing Up* participation. Young children's singing competences are relative to age and experience. If their pre-school musical experiences have been positive, they are more likely to enter formal schooling with successful participatory experience of singing in the home (Kirkpatrick, 1962; Williams et al., 2015; Soley and Spelke, 2016; Trehub and Gudmundsdottir, 2019) and to see singing as a natural cultural activity. Young children who have had a less rich or minimal pre-school experience of singing in the maternal culture will likely enter schooling needing more structured support—as is common in other areas of their development, such as the up to 50% of young children living in areas of deprivation who are reported to start school with identifiable Speech, Language and Communication Needs (SLCN) (Public Health England, 2020; Pitt and Welch, 2022). Nevertheless, the cluster analyses imply that more competent singers existed both within and without the *Sing Up* programme. This is confirmed in visual inspection of the standard deviations in the mean decimalised singing competence data in Figure 1 where some *Non-Sing Up* young children aged 6 to 8 years scored relatively high compared to their peers and, in some instances better than the children within the programme. Nevertheless, the inference from the data is that the programme was effective in promoting singing development, and—therefore—in creating proportionately more child singers with cluster 1 characteristics. From a school curriculum perspective, it is commonplace for singing activities to be undertaken in larger groups of children—whole classes and school assemblies. Consequently, the inference is that there are relatively few opportunities for individuals who are less developed in their singing to be supported unless the pedagogical approach is geared toward play, practice and mastery of a song's musical elements. Hedden (2012), for example, reviewed published literature from the previous twenty-five years on what effective singing pedagogy for children and young people might look like and advocated an approach which was sensitive to the underlying physiological development of the voice, as well as activities to promote vocal pitch matching and range extension. Such a range of activities accords with empirical data from systematic observations in diverse *Sing Up* Primary school classrooms as part of a wider grounded *Sing Up* evaluation of the possible criteria for successful singing (see Saunders et al., 2011). Examples of effective singing pedagogy included the vocal leader making the foci for the session explicit, providing vocal warm-ups, linking achievements to the lesson foci, providing guidance on how to improve (such as by using games, antiphoning, and call and response) and enabling children to be actively engaged with their voices throughout the session, including singing and opportunities for questions and answers.

Furthermore, our experience is that children who are less skilled singers are likely to recognize their lack of relative competency and to see themselves as on the margins in this musical activity. This, in turn, could impact on their general self-esteem in a school context if they feel that they are failing musically in front of their peers. Knight (2019), for example, reports on how adult “non-singers” (quotation marks in the orginal) made regular reference back to their negative experiences of singing in public in childhood and of how these experiences had stayed with them for decades into middle and late adulthood.

In terms of the pedagogical implications of the findings, other recent research in the UK and Australia affirms the potential for effective pedagogy to be linked to children's growing competency as singers. Two separate studies have each demonstrated that a structured programme which is focused on the mentoring of generalist (non-music specialist). Primary teachers by expert singers has both a positive impact on their professional music education competency to lead singing activities and, in turn, a related positive impact on their pupils' singing development (Barrett et al., 2020; Welch et al., 2020). The programme was focused on active participation in a specially selected song repertoire, made available both online through a Song Bank and also through a monthly magazine which provided support for teachers in terms of songs, vocal games, examples of success and background literature on how best to support singing development and a singing culture. As mentioned elsewhere, singing in English Primary schools is usually a whole class and whole school activity (in school assemblies). If you are a successful singer this is evidenced publicly. If you are less competent, this may be less significant in most of your peers are equally at the same phase of development. But if some are evidently more competent, you are likely to become aware of this through the comments of others, including well-meaning adults (Knight, 2019) and thus develop a negative attitude to singing and a negative singing identity. Furthermore, longitudinal evidence of young children's singing (Welch et al., 1996, 1997, 1998) demonstrated that a significant proportion of children aged 5–7 years have difficulty with pitch matching in song singing because their prime perceptual focus is on the lyrics. Children were much less accurate in their vocal pitching, especially boys, when lyrics were attached to the pitches (as in a song) compared with when the same children's singing was assessed without lyrics, i.e., on the musical features of a melody alone (*cf* Welch, 2016/2019). Effective singing pedagogy recognizes the need to enable children to explore and play with the ingredients of songs, including their key centers, both separately and in combination (Hedden, 2012; see, for example, the “inspire-music effective practice framework”—https://www.inspire-music.org/about-inspire-music).

Given the uneven numbers of young participants in the current study in each age group, it would be appropriate (a) to undertake more systematic comparative and longitudinal research into the nature of young children's singing behaviors and development, as well as (b) to explore how—as well as why—successful, current competency-level appropriate singing might be able to support other areas of development.

In conclusion, the data analyses suggest that the *Sing Up* programme tended to support the development of young participant children's singing abilities and also their attitudes toward singing as a part of their singing identities. There appears to be a positive symbiotic relationship between children's self-concept, sense of being socially included, and singing competency as evidenced by cluster 1 singers and which were found in both *Sing Up* and *Non-Sing Up* groupings. Pupils with a more positive singer identity tended to report more positive attitudes toward singing at school and other settings, higher levels of self-esteem and social integration, as well as more positive evaluations of their singing ability. At a time when young children have had their education and related attainment severely disrupted by the Covid-19 pandemic, particularly those in underserved communities (Rose et al., 2021), and with a reported increase in threats to children's mental health and well-being (Skripkauskaite et al., 2021), it is important to recognize that participation in high-quality singing activities can have a positive impact on young children across a range of areas, including singing ability, identity development, self-esteem and social integration.

## Data Availability Statement

The raw data supporting the conclusions of this article will be made available by the authors, without undue reservation.

## Ethics Statement

The studies involving human participants were reviewed and approved by the Institute of Education, University of London. Each child was provided with age appropriate information about the study for them and their parents. Written informed consent to participate in this study was provided by each school on behalf of the participants' legal guardian/next of kin. Participation was voluntary; children could withdraw at any time for any or no reason.

## Author Contributions

GW and IP conceived the article. GW led the research team, designed the study, and led the literature review. IP, JS, EH, and GW undertook fieldwork in participating schools across England. EH and JS created the database. IP led on the statistical analyses for this article and worked with GW on the shape and content of the article. All authors contributed to the article and approved the submitted version.

## Funding

This research was funded by the UK charity Youth Music as part of its overall evaluation of the National Singing Programme Sing Up in England.

## Conflict of Interest

The authors declare that the research was conducted in the absence of any commercial or financial relationships that could be construed as a potential conflict of interest.

## Publisher's Note

All claims expressed in this article are solely those of the authors and do not necessarily represent those of their affiliated organizations, or those of the publisher, the editors and the reviewers. Any product that may be evaluated in this article, or claim that may be made by its manufacturer, is not guaranteed or endorsed by the publisher.
